# Cordycepin augments the chemosensitivity of osteosarcoma to cisplatin by activating AMPK and suppressing the AKT signaling pathway

**DOI:** 10.1186/s12935-021-02411-y

**Published:** 2021-12-25

**Authors:** Hong-Bo Li, Jun-Kai Chen, Ze-Xin Su, Qing-Lin Jin, Li-Wen Deng, Gang Huang, Jing-Nan Shen

**Affiliations:** 1 Department of Musculoskeletal Oncology, The First Affiliated Hospital of Sun Yat-sen University, Guangzhou, 510080 China; 2 Guangdong Provincial Key Laboratory of Orthopedics and Traumatology, The First Affiliated Hospital of Sun Yat-sen University, Guangzhou, 510080 China

**Keywords:** Osteosarcoma, Cordycepin, Cisplatin, AMPK, AKT

## Abstract

**Background:**

Osteosarcoma is the most common primary bone tumor in children and adolescents. However, some patients with osteosarcoma develop resistance to chemotherapy, leading to a poor clinical prognosis. Hence, effective therapeutic agents that can improve the response to chemotherapy drugs to improve the prognosis of patients with osteosarcoma are urgently needed. Cordycepin has recently emerged as a promising antitumor drug candidate. This study aims to explore the effect of cordycepin in suppressing osteosarcoma in vivo and in vitro and the synergistic effect of cordycepin combined with cisplatin and to demonstrate the underlying molecular mechanism.

**Methods:**

CCK-8 assay was performed to investigate the inhibition effect of cordycepin combined with cisplatin in osteosarcoma cell lines. The colony formation and invasion abilities were measured by colony formation assay and Transwell assay. Osteosarcoma cells apoptosis was detected by flow cytometry. Western blot analysis were used to detect the expression of cell apoptosis-related proteins and AMPK and AKT/mTOR signaling pathway-related proteins. Finally, we performed the in vivo animal model to further explore whether cordycepin and cisplatin exert synergistic antitumor effects.

**Results:**

Notably, we found that treatment with cordycepin inhibited cell proliferation, invasion, and induced apoptosis in osteosarcoma cells in vitro and in vivo. Moreover, the combination of cordycepin and cisplatin led to marked inhibition of osteosarcoma cell proliferation and invasion and promoted osteosarcoma cell apoptosis in vitro and in vivo. Mechanistically, we demonstrated that cordycepin enhanced the sensitivity of osteosarcoma cells to cisplatin by activating AMPK and inhibiting the AKT/mTOR signaling pathway.

**Conclusions:**

In brief, this study provides comprehensive evidence that cordycepin inhibits osteosarcoma cell growth and invasion and induces osteosarcoma cell apoptosis by activating AMPK and inhibiting the AKT/mTOR signaling pathway and enhances the sensitivity of osteosarcoma cells to cisplatin, suggesting that cordycepin is a promising treatment for osteosarcoma.

## Background

Osteosarcoma is the most common primary malignant bone tumor in children and adolescents and accounts for approximately 55% of all primary malignant bone tumors [1]. In the last two decades, due to the combination of surgery and chemotherapy, the 5-year survival rate has reached 60–70% in patients with localized tumors [2]. However, standard adjuvant/neoadjuvant chemotherapy does not have robust antineoplastic effects in some osteosarcoma patients [3, 4], and a considerable number of patients develop drug resistance to current chemotherapy regimens [5]. Clinically, a significant number of patients are either insensitive to chemotherapy or susceptible to chemotherapy resistance under current chemotherapy regimens [5]. Therefore, novel and effective drugs for the better management of osteosarcoma patients are urgently needed.

Cordycepin, also known as 3-deoxyadenosine, a nucleoside analog, is a natural product isolated and purified from and the main active component of *Cordyceps militaris*. Cordycepin is a nucleoside drug and purine alkaloid that exhibits numerous biological effects, such as on anti-inflammatory Response [6], anti-immunological [7], and antitumor effects [8, 9]. Many studies have shown that cordycepin has significant antitumor effects, promoting cell apoptosis and suppressing cell proliferation and invasion and tumor metastasis through various signaling pathways [9–12]. However, the inhibitory effect of cordycepin on osteosarcoma has not been reported, and the molecular mechanisms of its anti-tumor activity remain unclear.

Cisplatin is a classic chemotherapeutic agent recommended by the National Comprehensive Cancer Network (NCCN) guidelines for the treatment of osteosarcoma via the MAP regimen [13]. Cisplatin mainly cross-links with interchain DNA in cells, resulting in DNA loss and inducing cytotoxicity [14]. Drug resistance to cisplatin, which greatly weakens the efficacy of cisplatin and prevents it from improving the prognosis of patients with osteosarcoma, occurs frequently in the clinic. Therefore, it is very important to identify effective therapeutic agents that can improve the response to chemotherapy drugs to improve the prognosis of patients with osteosarcoma.

In this study, we aimed to explore the effect of cordycepin in suppressing osteosarcoma in vivo and in vitro and to determine its antitumor molecular mechanism. In addition, we aimed to explore the synergistic effect of cordycepin and cisplatin in the treatment of osteosarcoma. The results of this study may provide evidence that cordycepin is a promising agent for the treatment of osteosarcoma.

## Materials and methods

### Reagents

Cordycepin and cisplatin were purchased from Sigma (St. Louis, Missouri).

### Cell culture

The osteosarcoma cell lines U2OS, SAOS2, MNNGHOS, 143B, MG63, SJSA1, and osteoblast cell (hFOB) were obtained from American Type Culture Collection (ATCC). The U2OS/MTX300 (U2R) cell line, a methotrexate-resistant derivative of the U2OS human osteosarcoma cell line, was kindly provided by Dr. M. Serra (Istituti Ortopedici Rizzoli, Bologna, Italy). All cell lines were cultured in Dulbecco’s modified Eagle’s medium (DMEM, Gibco, Grand Island, NY, USA) supplemented with 10% fetal bovine serum (Gibco, Grand Island, NY, USA), penicillin (100 U/mL) and streptomycin (100 µg/mL) according to the instructions of ATCC.

### CCK-8 assay

A total of 2000 osteosarcoma cells were plated in 96-well plates and exposed to different concentrations of cordycepin and/or cisplatin at 37 °C in a 5% CO_2_ humidified incubator. After the indicated times, 10 µL CCK-8 solution was added to each well, and the plates were incubated at 37 °C for 2 h. The absorbance was determined at 450 nm using a microplate reader. The proliferative inhibition rate was calculated as follows: proliferative inhibition rate = (1 − experimental group/control group) × 100%. The IC_50_ value was calculated by SPSS 20.0 software using non-linear regression analysis.

The combination index (CI) was calculated by isobologram analysis based on the Chou-Talalay method. The two drugs interactions were categorized into synergism (CI < 1), antagonism (CI > 1), and additive effect (CI = 1), respectively.

### Colony formation assay

Cells were plated in 6-well plates in 2 mL DMEM containing 10% fetal bovine serum (500 cells/well). The 6-well plates were placed in a 5% CO2 humidified incubator at 37 °C and incubated with or without cordycepin and/or cisplatin. After 14 days, the supernatant was removed. The cells were washed with PBS, fixed with methanol, and stained with crystal violet. After staining with crystal violet, colonies containing > 50 cells were counted.

### Analysis of apoptosis by annexin V/PI staining

Cells were plated in 6-well plates and treated with different concentrations of cordycepin and/or cisplatin for 48 h. The cells were subsequently collected, washed with PBS twice, and resuspended in binding buffer (500 µL). Then, 5 µL Annexin V-FITC and 5 µL PI were added to the buffer, the solution as mixed well, and the cells were incubated at room temperature in the dark for 15 min. The cells were analyzed by flow cytometry.

### Transwell assay

Transwell cell culture chambers with 8 mm microporous filter (Corning, Cat.no.353,097) with a precoating of extracellular matrix coating (BD Biosciences) were been taken out and placed in 24 well plate. 200 µL of cell suspension in serum-free DMEM were add to the Transwell cell culture chambers of 24-well plates, and 500 µL of DMEM with 20%FBS was added to the 24 well plate. After 24 h of incubation at 37 °C, the cells in the Transwell cell culture chambers were stained with 0.25% crystal violet. The cells remaining in the upper Transwell chamber were removed, and those that migrated to the lower chamber were photographed and counted. Each Transwell chamber counts five fields randomly (magnification, ×100).

### Western blot analysis

Proteins were extracted from cells with RIPA buffer containing a protease inhibitor and phosphatase inhibitor (Roche), and the cell debris was removed by centrifugation (14,000×*g*, 30 min). The protein concentration was determined by a BCA protein assay kit. Loading buffer was added to equal amounts of protein, and the samples were boiled for 10 min, resolved by SDS-PAGE and transferred to polyvinylidene fluoride membranes. The membranes were blocked in 5% skim milk for 1 h at room temperature and incubated with a primary antibody against Bax (1:1000, #5023S, CST), Bcl-2 (1:1000, #15071S, CST), Cleaved PARP (1:1000, #9661S, CST), MMP-2 (1:1000, #40994S, CST), MMP-9 (1:1000, #13667S, CST), p-AMPK (Thr172) (1:1000, #ab133448, Abcam), AMPK (1:1000, #ab32047, Abcam), p-AKT (Ser473) (1:2000, #4060S, CST), AKT (1:1000, #4685S, CST), p-mTOR (Ser2448) (1:1000, #5536S, CST), mTOR (1:1000, #2983S, CST), p-p70S6K (Thr389) (1:1000, #97596S, CST), or p70S6K (1:1000, #2708S, CST), GAPDH (1:5000, 10494-1-AP, Proteintech,) overnight at 4 °C. The membranes were incubated with secondary antibodies at room temperature for 1 h after being washed three times with TBST. Finally, the membranes were subjected to chemiluminescence using ECL.

### Immunohistochemistry

Paraffin-embedded specimens fixed in formalin were collected. The paraffin sections were placed in dimethylbenzene, dimethylbenzene, 100% ethanol, 100% ethanol, 95% ethanol, 90% ethanol, 80% ethanol and 70% ethanol in succession for dewaxing. After dewaxing, 3% H_2_O_2_ was added for 10 min to block endogenous catalase activity. Citric acid buffer was added, and the sections were boiled for 3 min, cooled to room temperature, and then boiled again. When the sections were cooled to room temperature, they were blocked with goat serum for 1 h at room temperature and incubated with a primary antibody (Ki-67, 1:100, #9449, CST) overnight at 4 °C. The slides were incubated with secondary antibodies at room temperature for 1 h after being washed three times with PBS. Then, they were incubated with streptavidin-biotin complex (SABC) at 37 °C for 30 min and developed with DAB for 10 min. Finally, the sections were counterstained with hematoxylin, dehydrated and sealed.

### Animal studies

All female BALB/c nude mice (4–5 weeks old) were purchased from GemPharmatech Co., Ltd. The animal experiment was approved by the Institutional Review Board of The First Affiliated Hospital of Sun Yat-sen University, and the animals were raised in the Animal Experiment Center of the First Affiliated Hospital of Sun Yat-sen University. After the mice were anesthetized with isoflurane, 1 × 10^6^ suspended 143B cells in 20 µL were injected into the proximal tibia through the anterior tuberosity. The nude mice were randomly divided into the following four groups after approximately 14 days, when the tumor volume reached approximately 200 mm^3^, and administered drugs by intraperitoneal (i.p.) injection: the control group, cordycepin treatment group (40 mg/kg, every day), cisplatin treatment group (5 mg/kg, every 3 days), and the combined treatment group [cordycepin (40 mg/kg, every day) and cisplatin (5 mg/kg, every 3 days)]. The mice were monitored every 3 days. The size of the tumors and the weights of the mice were recorded. The size of the tumors was measured in two perpendicular dimensions (D1 and D2). The tumor volume was calculated using the formula V = 4/3π [1/4 (D1 + D2)]^2^, as described previously [15]. At the end of the experiment, the mice were euthanized by cervical dislocation. H&E staining of nude mouse tissue was performed according to standard procedures.

### Statistical analyses

All statistical analyses were performed using SPSS version 20.0 software. The data are expressed as the mean ± SD of three independent experiments, and data from one representative experiment are shown. *P* values were determined by Student’s *t*-test or one-way ANOVA, as indicated in the legends. *P* < 0.05 was considered statistically significant.

## Results

### Cordycepin inhibits osteosarcoma cell viability

**Fig. 1:**
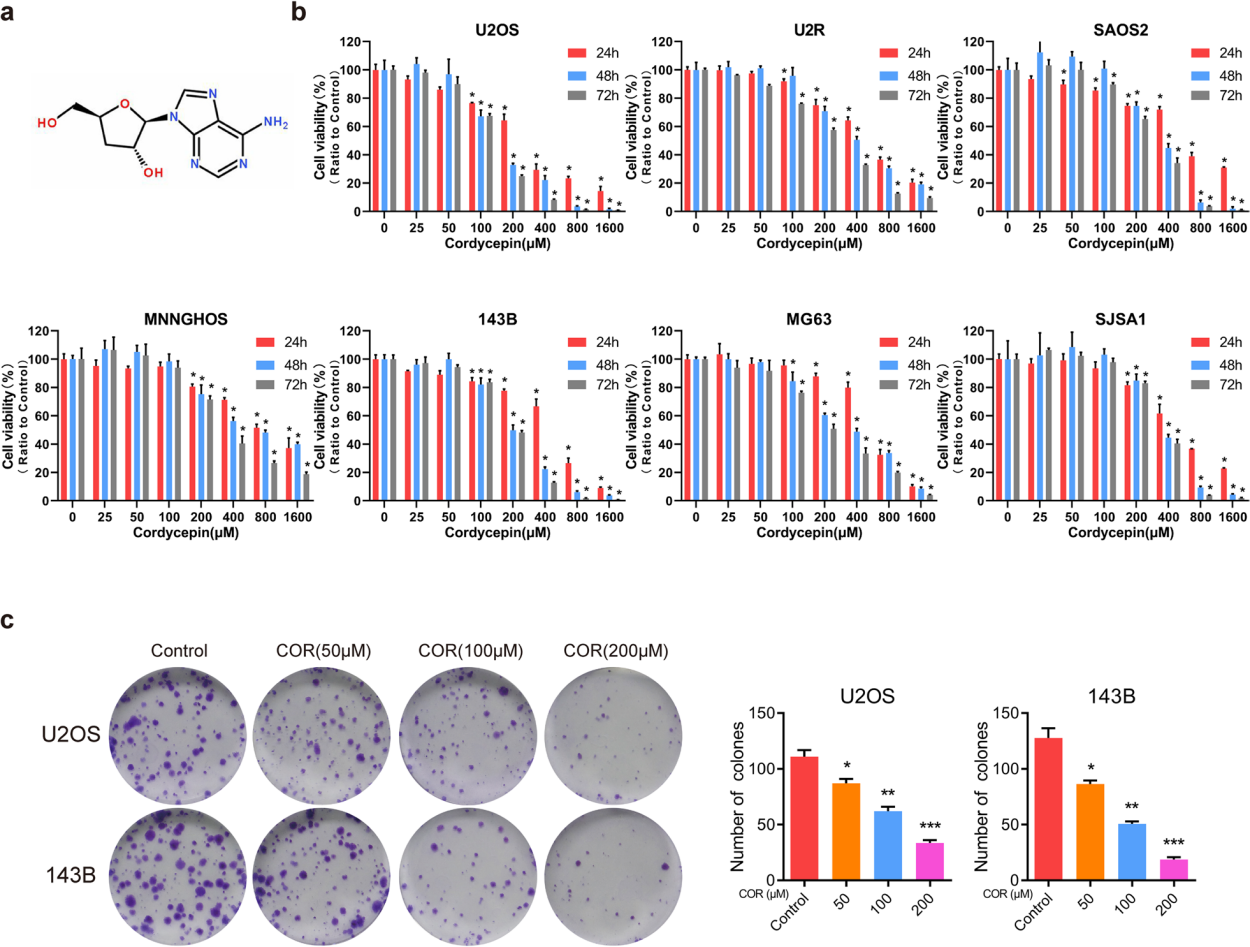
Cordycepin inhibits osteosarcoma cell viability. **a** The chemical structure of cordycepin; **b** CCK-8 assay showing cordycepin inhibits osteosarcoma cell (U2OS, U2R, SAOS2, MNNGHOS, 143B, MG63, and SJSA1) viability in a time- and dose-dependent manner. Osteosarcoma cells were treated with various concentrations of cordycepin for 24, 48, and 72 h; **c** Cordycepin reduces colony formation of osteosarcoma cells. Colony-formation abilities of U2OS and 143B osteosarcoma cells were examined after cordycepin treatment for 14 days. Representative images and quantification of the colony formation results are shown. The data are expressed as the mean ± SD of three independent experiments. *p < 0.05; **p < 0.01; ***p < 0.001, by Student’s *t*-test

The chemical structure of cordycepin is shown in Fig. 1a. Because cordycepin has an obvious antitumor effect on a variety of tumors, we treated U2OS, U2R, SAOS2, MNNGHOS, 143B, MG63, and SJSA1 osteosarcoma cells with cordycepin at various concentrations for 48 or 72 h to investigate the toxic effect of cordycepin on these cells. The CCK-8 assay showed that compared to control treatment, cordycepin treatment inhibited the proliferation of osteosarcoma cells, resulting in dose- and time-dependent growth inhibition (Fig. 1b). The IC_50_ values of cordycepin at 48 and 72 h were 155.1 µM and 131.6 µM in U2OS cells, 372.1 µM and 346.3 µM in SJSA1 cells, 346.0 µM and 275.9 µM in SAOS2 cells, 208.5 µM and 191.8 µM in 143B cells, 443.6 µM and 237.4 µM in U2R cells, 775.7 µM and 381 µM in MNNGHOS cells, and 367.5 µM and 231.6 µM in MG63 cells. Additionally, we performed a colony formation assay to assess the inhibitory effect of cordycepin on U2OS and 143B osteosarcoma cells. The results revealed that treatment with cordycepin inhibited the colony formation ability of U2OS and 143B cells after incubation with cordycepin for 14 days (Fig. 1c). There was a significant dose-dependent reduction in the size and number of colony-forming cells in the cordycepin treatment group compared to the control group. These results indicate that cordycepin effectively inhibits osteosarcoma cell viability.

### Cordycepin induces osteosarcoma cell apoptosis

**Fig. 2:**
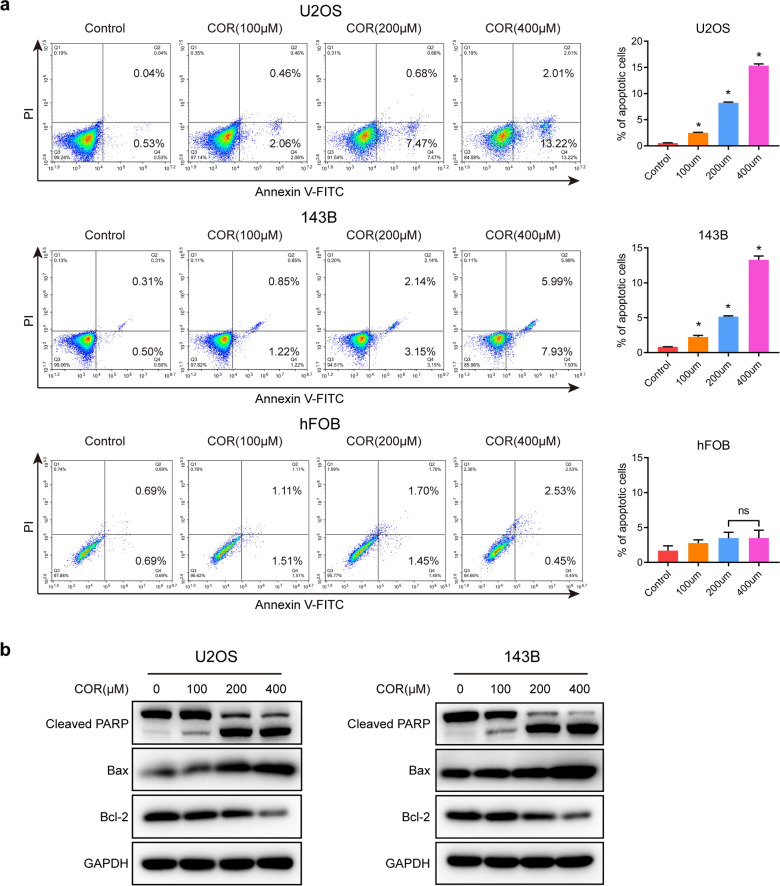
Cordycepin induces osteosarcoma cell apoptosis. **a** Annexin V/PI staining of U2OS, 143B osteosarcoma cells, and normal osteoblast cell hFOB treated with or without cordycepin at 100, 200, and 400 µM for 48 h was detected and analyzed by flow cytometry. Representative images and quantification of the flow cytometry analysis results are shown; **b** Western blotting analysis of the expression levels of cleaved PARP, Bax, and Bcl-2 in both U2OS and 143B cells following a 48-h treatment with cordycepin at each designated concentration. The data are expressed as the mean ± SD of three independent experiments. *p < 0.05; **p < 0.01; ***p < 0.001, by Student’s *t*-test

Above, we demonstrated that cordycepin can significantly inhibit the proliferation of osteosarcoma cells. Next, we explored whether cordycepin has an effect on the apoptosis of osteosarcoma cells. We treated U2OS and 143B cells with different concentrations of cordycepin for 48 h and then collected the cells for analysis of apoptosis by Annexin V/PI staining. The results revealed that the proportion of cells labeled with Annexin V/PI in the cordycepin treatment group was increased compared to that in the control group. In contrast, cordycepin induced a limited apoptosis effect on normal osteoblast cells hFOB (Fig. 2a). Our results indicated that cordycepin significantly induced apoptosis of osteosarcoma cells in a dose-dependent manner. To further confirm these results, we measured the expression of apoptosis-related proteins in U2OS and 143B osteosarcoma cells treated with cordycepin for 48 h by western blotting. The results showed that the expression of the proapoptotic protein Bax was dose-dependently increased while that of the antiapoptotic protein Bcl-2 was dose-dependently decreased after treatment with cordycepin. Furthermore, we found that the expression of Cleaved PARP was increased in the cordycepin treatment group (Fig. 2b). Therefore, our results suggest that cordycepin induces apoptosis in osteosarcoma cells.

### Cordycepin enhances the sensitivity of osteosarcoma cells to cisplatin to inhibit osteosarcoma cell proliferation and induce osteosarcoma cell apoptosis

**Fig. 3:**
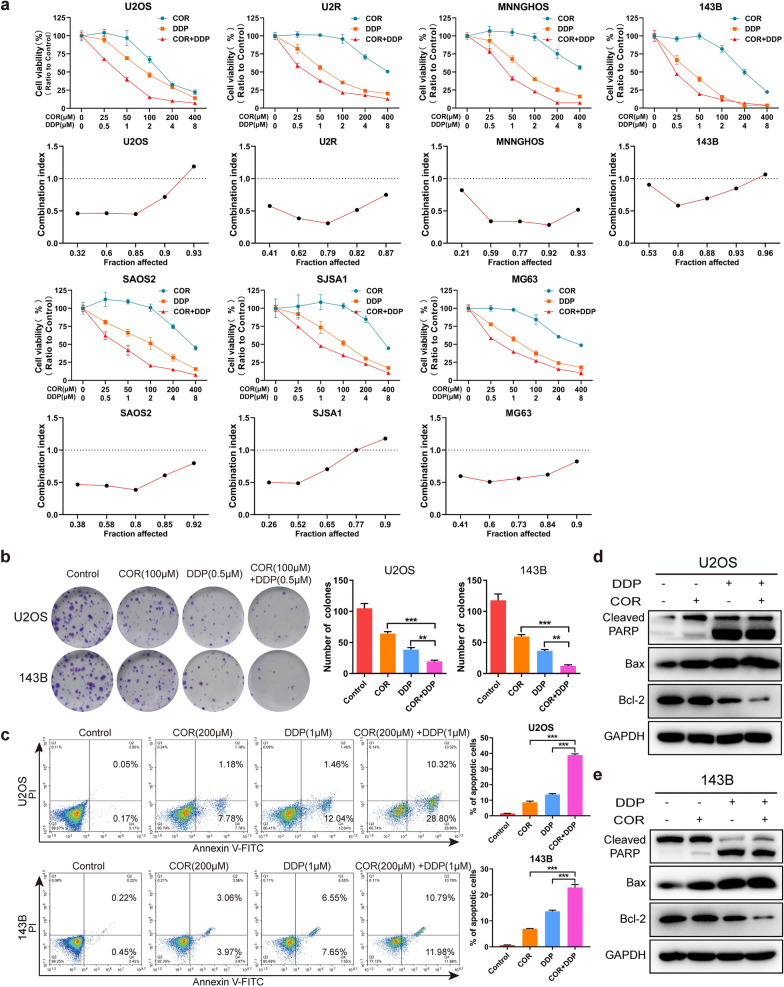
Cordycepin enhances the sensitivity of osteosarcoma cells to cisplatin to inhibit osteosarcoma cell proliferation and induce osteosarcoma cell apoptosis. **a** CCK-8 assay showing the viability of osteosarcoma cell (U2OS, U2R, SAOS2, MNNGHOS, 143B, MG63, and SJSA1) treated with cordycepin and cisplatin at each designated concentration for 48 h. The fraction affected (Fa)-combination index (CI) plots showing that cordycepin exhibited an apparently synergistic effect when combined with cisplatin (CI < 1); **b** Colony formation experiments showing the colony numbers of U2OS and 143B cells following treatment with cordycepin (100 µM), cisplatin (0.5 µM), or their combination (100 µM cordycepin and 0.5 µM cisplatin). Representative images and quantification of the colony formation results are shown; **c** Annexin V/PI staining of osteosarcoma cells (U2OS, 143B) treated with cordycepin (200 µM), cisplatin (1 µM), or their combination (200 µM cordycepin and 1 µM cisplatin) for 48 h was detected and analyzed by flow cytometry. Representative images and quantification of the flow cytometry analysis results are shown; **d**, **e** Western blotting analysis of the expression levels of cleaved PARP, Bax, and Bcl-2 in both U2OS and 143B cells following a treatment with cordycepin (200 µM), cisplatin (1 µM), or their combination for 48 h. The combination therapy of both cordycepin and cisplatin enhanced the apoptosis of osteosarcoma cells, compared to treatment with each drug alone. The data are expressed as the mean±SD of three independent experiments. *p < 0.05; **p < 0.01; ***p < 0.001, by Student’s *t*-test

In our previous studies, we confirmed that both cordycepin and cisplatin can inhibit the proliferation of osteosarcoma cells in a dose-dependent manner. To investigate whether cordycepin can enhance the inhibitory effect of cisplatin on osteosarcoma cell proliferation, we performed a CCK-8 assay using U2OS, U2R, SAOS2, MNNGHOS, 143B, MG63, and SJSA1 cells treated with cordycepin and/or cisplatin at different concentrations for 48 h. The results showed that the therapeutic effect of the combination of cordycepin and cisplatin was enhanced compared with that cordycepin or cisplatin alone (Fig. 3a). To determine the synergistic, additive, or antagonistic effects of combination therapy, we calculated the combination index (CI). The results suggested that cordycepin had a synergistic effect with cisplatin (CI < 1) in inhibiting osteosarcoma cell proliferation following treatment for 48 h (Fig. 3a). Colony formation experiments also showed that combination therapy with both cordycepin (100 µM) and cisplatin (0.5 µM) for 14 days inhibited the colony formation ability of U2OS and 143B cells (Fig. 3b).

To explore whether cordycepin combined with cisplatin can affect the apoptosis of osteosarcoma cells, we collected cells treated with cordycepin (200 µM) and/or cisplatin (1 µM) at different concentrations for 48 h and detected cell apoptosis by Annexin V/PI staining. Apoptosis was induced in osteosarcoma cells treated with the combination of cordycepin and cisplatin compared to cells treated with cordycepin or cisplatin alone (Fig. 3c). Western blotting also showed that the expression levels of Cleaved PARP, and Bax were significantly increased by the combination of cordycepin and cisplatin compared to either drug alone. Moreover, we found that the expression of the antiapoptotic protein Bcl-2 was significantly decreased in U2OS and 143B osteosarcoma cells treated with both cordycepin and cisplatin compared to those treated with either drug alone (Fig. 3d, e). In summary, the combination of cordycepin and cisplatin markedly promotes osteosarcoma cell apoptosis.

### Cordycepin increases osteosarcoma cell sensitivity to cisplatin by activating the AMPK signaling pathway and suppressing the AKT signaling pathway

**Fig. 4:**
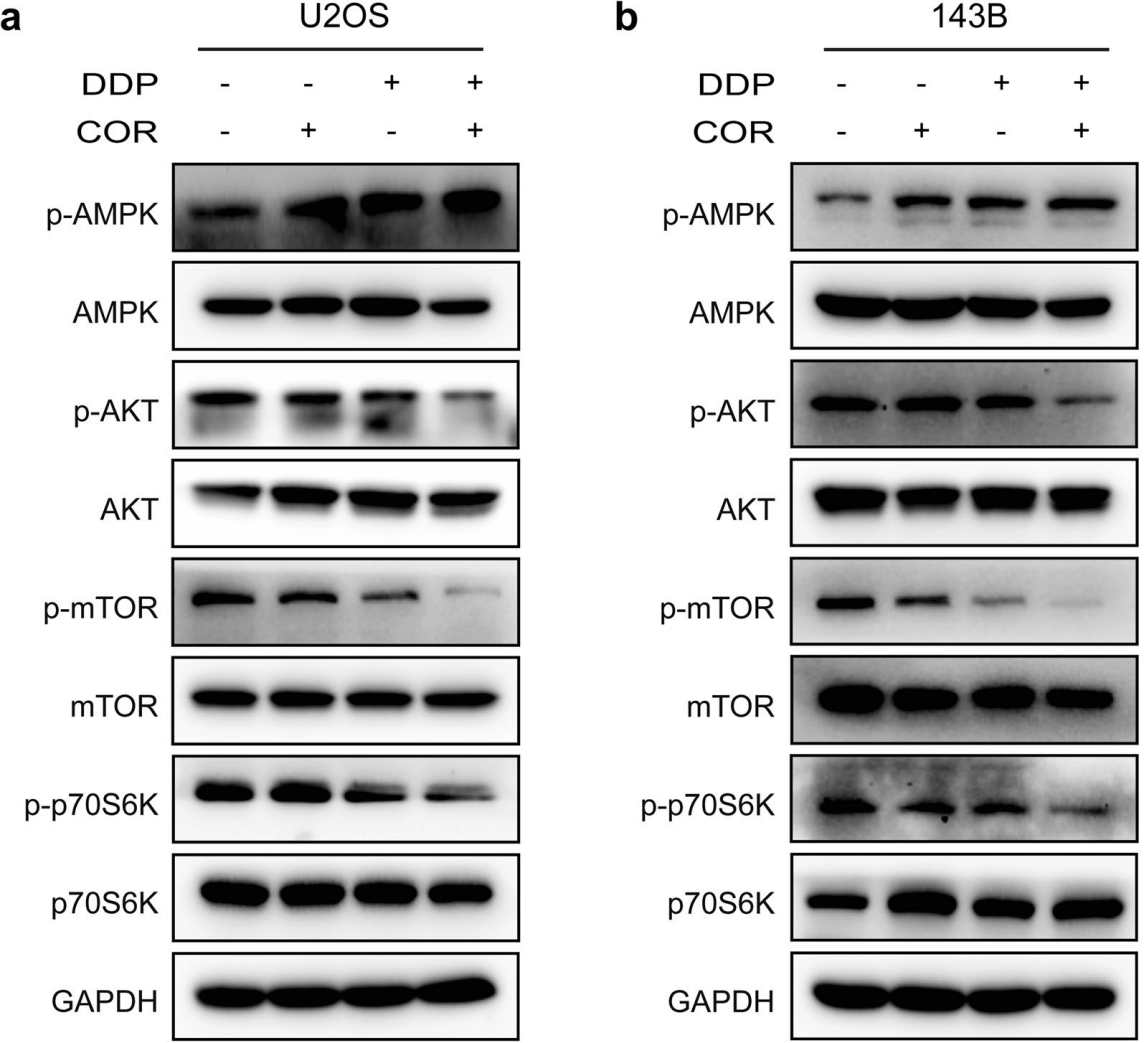
Cordycepin increases osteosarcoma cell sensitivity to cisplatin by activating the AMPK signaling pathway and suppressing the AKT signaling pathway. **a**, **b** Western blotting analysis showing the protein expression levels of p-AMPK, p-AKT, p-mTOR, and p-p70S6K in U2OS and 143B osteosarcoma cells following a treatment with cordycepin (200 µM), cisplatin (1 µM), or their combination for 48 h

It has been reported that cordycepin may inhibit intracellular lipid accumulation through activation of AMPK via interaction with the γ1 subunit and might act as a novel AMPK activator for the treatment of hepatic steatosis, inflammation, liver injury, and a variety of tumors by activating the AMPK signaling pathway [12, 16, 17]. To determine whether cordycepin inhibits osteosarcoma cell growth through the AMPK pathway, we performed western blotting and found that the expression of p-AMPK was upregulated after treatment with cordycepin for 48 h. We also found that activation of AMPK was enhanced in the group treated with the combination of cordycepin and cisplatin compared to the group treated with cordycepin or cisplatin alone (Fig. 4a, b).

The serine/threonine kinase AKT, also known as protein kinase B (PKB), is a central node of signaling downstream of growth factors and is correlated with tumorigenicity [18]. Some reports indicate that activation of the AKT/mTOR signaling pathway plays an important role in the progression of osteosarcoma and cisplatin resistance [19–21]. To confirm whether the effect of cordycepin on the inhibition of osteosarcoma growth is mediated by suppression of the AKT/mTOR signaling pathway, we treated osteosarcoma cells with cordycepin for 48 h and performed western blotting. The results verified that the expression of p-AKT, p-mTOR, and p-p70S6K as downregulated after treatment with cordycepin. It has been reported that activation of the Akt pathway induces cisplatin resistance by inhibiting propagation of the DNA damage signal to the apoptotic machinery [14, 22]. To further investigate whether cordycepin increases the sensitivity of osteosarcoma cells to the inhibitory effects of cisplatin by inhibiting AKT signaling, we treated osteosarcoma U2OS and 143B cells with both cordycepin and cisplatin for 48 h and performed western blotting. The results showed that the expression levels of p-AKT, p-mTOR, and p-p70S6K were significantly decreased in the groups treated with the combination of cordycepin and cisplatin compared to the control group and the groups treated with either drug alone (Fig. 4a, b). Altogether, our results indicate that cordycepin augments the chemosensitivity of osteosarcoma cells to cisplatin by activating AMPK and suppressing the AKT/mTOR signaling pathway.

### Cordycepin and cisplatin synergistically inhibit osteosarcoma cell invasion by downregulating MMP-2 and MMP-9 expression

**Fig. 5:**
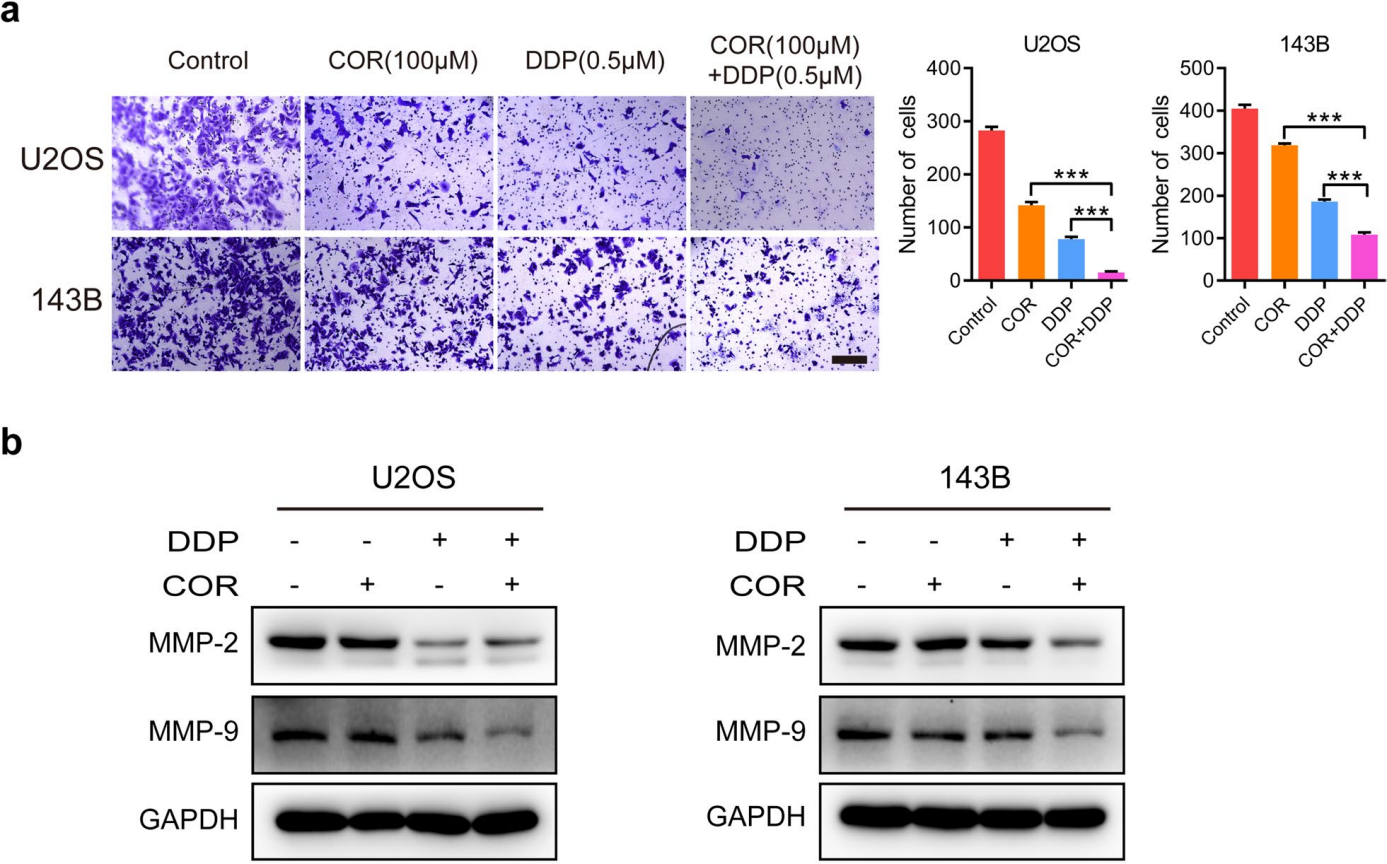
Cordycepin and cisplatin synergistically inhibit osteosarcoma cell invasion by downregulating MMP-2 and MMP-9 expression. **a** Transwell assay showed that the invasion abilities of U2OS and 143B cells were inhibited following treatment with cordycepin (100 µM), cisplatin (0.5 µM), or their combination for 48 h. Representative images and quantification of the transwell results are shown; **b** Western blotting analysis showing the protein expression levels of MMP-2 and MMP-9 in U2OS and 143B cells treated with vehicle (Control), cordycepin (200 µM), cisplatin (1 µM), or their combination for 48 h. The data are expressed as the mean ± SD of three independent experiments. *p < 0.05; **p < 0.01; ***p < 0.001, by Student’s *t*-test, scale bars = 100 μm

Our previous results showed that cordycepin can enhance sensitivity of osteosarcoma cells to cisplatin-mediated inhibition of cell proliferation and induction of cell apoptosis. Thus, we performed Transwell assays to determine whether cordycepin enhances cisplatin-mediated inhibition of osteosarcoma cell invasion. We found that cordycepin significantly reduced the cellular invasion ability of U2OS and 143B cells. Cordycepin significantly promoted the cisplatin-mediated inhibition of the cellular invasion of osteosarcoma cells (Fig. 5a). In addition, western blotting revealed that the expression levels of MMP-2 and MMP-9 were decreased in osteosarcoma cells treated with both cordycepin and cisplatin for 48 h compared to those in the control group and those treated with either drug alone (Fig. 5b). The above results indicate that combination therapy with both cordycepin and cisplatin synergistically inhibits osteosarcoma cell invasion.

### Cordycepin and cisplatin synergistically inhibit the growth of osteosarcoma xenografts in vivo

**Fig. 6:**
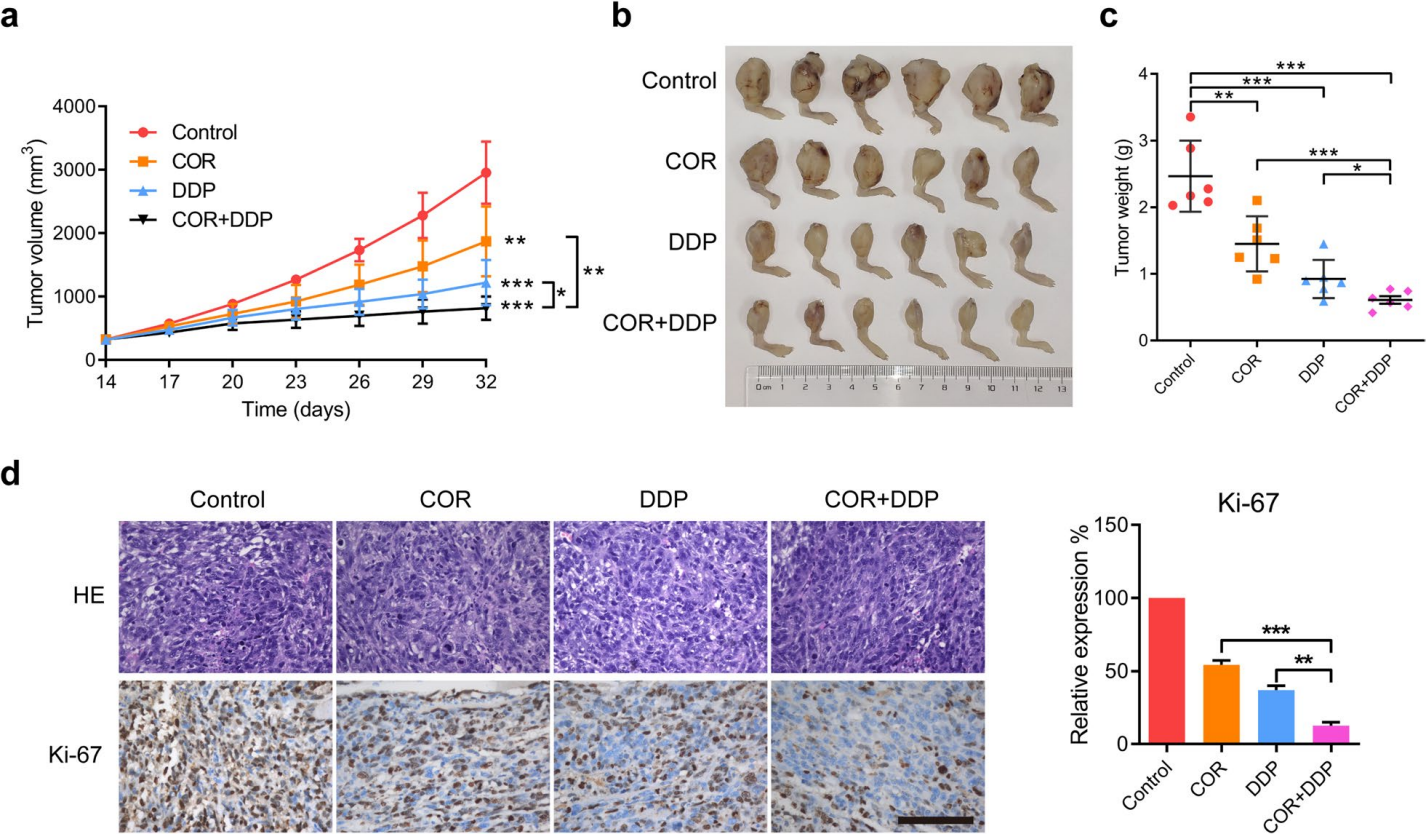
Cordycepin and cisplatin synergistically inhibit the growth of osteosarcoma xenografts in vivo. **a**–**c** Examination of tumor volumes and weight to evaluate the effect of different treatments (Vehicle, cordycepin 40 mg/kg, cisplatin 5 mg/kg, or the combined treatment with both cordycepin 40 mg/kg and cisplatin 5 mg/kg) on 143B cells in a xenograft model. The tumor volume of all groups was measured every 3 days. The tumor and the statistical analysis of tumor volumes in different groups was shown. Data represent the mean ± SD of tumor volume and weight of six mice; **d** Immunohistochemistry staining of Ki-67 in mouse tumor samples to examine the inhibitory effect on osteosarcoma cell proliferation by cordycepin or/and cisplatin treatment. Combined treatment of cordycepin and cisplatin had a significant decrease in the expression of Ki-67 *in vivo*. The data are expressed as the mean±SD of three independent experiments. *p < 0.05; **p < 0.01; ***p < 0.001, by Student’s *t*-test, scale bars=100 μm

The above experimental evidence shows that combined treatment with cordycepin and cisplatin synergistically inhibits the growth of osteosarcoma *in vitro*. To further explore whether cordycepin and cisplatin exert synergistic antitumor effects *in vivo*, we subcutaneously injected 143B cells into nude mice. When the tumor volume reached approximately 200 mm^3^ after ten days, the nude mice were randomly subdivided into the following four groups and administered different drugs: the control group, cordycepin treatment group (40 mg/kg), cisplatin treatment group (5 mg/kg), and combined treatment group [cordycepin (40 mg/kg) and cisplatin (5 mg/kg)]. The results showed that tumor growth was significantly reduced in the cordycepin treatment group and cisplatin treatment group compared to the control group, as reflected by tumor volume and weight (Fig. 6a–c). The growth of osteosarcoma xenografts in mice was significantly inhibited in the cordycepin (40 mg/kg) and cisplatin (5 mg/kg) treatment group compared to the control group and the groups treated with either drug alone. Moreover, immunohistochemical analysis of mouse tumor samples revealed that combined treatment with cordycepin and cisplatin significantly decreased the expression of Ki-67 (Fig. 6d). In summary, cordycepin and cisplatin synergistically inhibit the growth of osteosarcoma xenografts *in vivo*.

## Discussion

Osteosarcoma is the most common primary bone malignancy in children and adolescents, accounting for approximately 55% of all primary bone malignancies [1]. The survival rate of patients with osteosarcoma treated by surgery alone is approximately 15–17%, and some osteosarcoma patients do not show significant antitumor effects after standard adjuvant/neoadjuvant chemotherapy treatment [3, 4]. Clinically, some osteosarcoma patients are insensitive to chemotherapy or are prone to chemotherapy resistance [5]. Therefore, it is very important to identify effective therapeutic agents that can improve the response to chemotherapy drugs to improve the prognosis of patients with osteosarcoma.

Cordycepin has been reported to have significant antitumor effects, including apoptosis-inducing and cell proliferation-, invasion-, and tumor metastasis-inhibiting effects [9–12]. However, the inhibitory effect of cordycepin on osteosarcoma has not been reported. In this study, we confirmed the anti-osteosarcoma effect of cordycepin through in vitro and in vivo experiments. We demonstrated that cordycepin significantly induced apoptosis of osteosarcoma cells and significantly inhibited osteosarcoma cell proliferation and invasion. In addition, the inhibitory effect of cordycepin was time- and dose-dependent. We found that cordycepin significantly reduced the protein expression levels of Bcl-2, MMP-2, and MMP-9 and induce increased the expression levels of Bax, and Cleaved PARP in osteosarcoma cells. Mechanistically, we found that cordycepin mediated the activation of AMPK and inhibited the AKT signaling pathway, thus playing a role in inhibiting the growth of osteosarcoma cells.

Cisplatin is a classic chemotherapy drug recommended by the NCCN guidelines for the treatment of osteosarcoma via the MAP regimen [13]. However, drug resistance to chemotherapy, which greatly weakens drug efficacy and prevents improvements in the prognosis of patients with osteosarcoma, often occurs during clinical use. Therefore, it is particularly important to find suitable drug combinations to improve the cytotoxicity and curative effect of chemotherapy drugs to improve the prognosis of patients with osteosarcoma. In this study, we found that cordycepin combined with cisplatin had a synergistic effect, significantly reducing resistance to cisplatin and increasing chemotherapy sensitivity. The combination of cordycepin and cisplatin showed a stronger cell-killing effect than either drug individually and reduced colony formation and cell invasion abilities. *In vivo* experiments also showed that the combination of cordycepin and cisplatin had a significant inhibitory effect on tumors that was more obvious than that of cordycepin or cisplatin alone, significantly reducing the tumor volume. Our study shows that cordycepin can significantly enhance sensitivity to cisplatin, providing a new therapeutic strategy for the drug treatment of osteosarcoma.

The AMPK pathway plays an important role in the regulation of cell growth and metabolism as a cellular energy regulator and regulates tumor growth and proliferation [23–25]. Cordycepin has been comfirm to inhibit drug-resistance non-small cell lung cancer progression through activating AMPK signaling pathway [26]. Cordycepin might be a novel AMPK activator that binds the α1 and γ1 subunits near the autoinhibitory domain of AMPK [17, 27]. Our study revealed that cordycepin mediated an increase in AMPK phosphorylation, thereby inhibiting the proliferation of osteosarcoma cells and that the expression of phosphorylated AMPK increased more significantly when cordycepin was used in combination with cisplatin than when it was administered alone. The abnormal activation of AKT/mTOR signaling pathway can accelerate the proliferation of tumor cells, enhance resistance to apoptosis, and promote tumor invasion and metastasis [28–30]. Activation of the AKT signaling pathway is also widely believed to be associated with the resistance of tumor cells to chemotherapy [31–33]. It has been reported that activation of the AKT signaling pathway is likely to inhibit the transmission of DNA damage signals to the apoptosis machinery, resulting in the loss of DNA damage recognition and the induction of cisplatin resistance [14]. Recent studies have shown that the PI3K/AKT/mTOR signaling pathway is widely activated in osteosarcoma cells, especially in the drug resistant osteosarcoma cells [34]. In our study, we found that the phosphorylation levels of AKT signaling pathway-related proteins were decreased after treatment with cordycepin or cisplatin. More importantly, the combination of cordycepin and cisplatin had a more significant inhibitory effect on the AKT and mTOR pathways. Western blotting showed that the expression levels of p-AKT, p-mTOR and p-P70S6K were significantly decreased in the group treated with both cordycepin and cisplatin compared with the control group and the groups treated with either drug alone. P70S6K is a ribosome 40 S small subunit S6 protein kinase and an important downstream target regulated by mTOR, which plays important roles in cell proliferation, cell survival [35]. Our results showed that the combination of cordycepin and cisplatin significantly reduced the phosphorylation level of p70S6K. Emerging evidence indicates that MMPs act a pivotal part in various tumor invasion and metastasis [36]. Among them, MMP-2 and MMP-9 play a key role in initiating osteosarcoma cell metastasis and associate with poor response to chemotherapy in osteosarcoma [37]. Studies have shown that MMP-2 and MMP-9 are involved in AKT/mTOR-mediated invasion and metastasis of osteosarcoma cells [38]. Our study revealed that cordycepin and cisplatin synergistically inhibited osteosarcoma cell invasion and invasion by downregulating MMP-2 and MMP-9 expression. These results indicate that cordycepin enhances the sensitivity of osteosarcoma cells to cisplatin by activating AMPK and inhibiting the AKT/mTOR signaling pathway.

## Conclusions

In summary, this study provides comprehensive evidence that cordycepin inhibits the growth and apoptosis of osteosarcoma cells by activating AMPK and inhibiting the AKT/mTOR signaling pathway and enhances the sensitivity of osteosarcoma cells to cisplatin, suggesting that cordycepin is a promising novel treatment for osteosarcoma.

## Data Availability

All data generated or analyzed are submitted to the journal.

## Abbreviations

COR: Cordycepin
DDP: Cisplatin
HE: Hematoxylin and eosin
IHC: Immunohistochemical
NCCN: National Comprehensive Cancer Network
MAP regimen: High-dose methotrexate, cisplatin, and doxorubicin.
